# Combining [^11^C]-AnxA5 PET Imaging with Serum Biomarkers for Improved Detection in Live Mice of Modest Cell Death in Human Solid Tumor Xenografts

**DOI:** 10.1371/journal.pone.0042151

**Published:** 2012-08-01

**Authors:** Qing Cheng, Li Lu, Jonas Grafström, Maria Hägg Olofsson, Jan-Olov Thorell, Erik Samén, Katarina Johansson, Hanna-Stina Ahlzén, Sharon Stone-Elander, Stig Linder, Elias S. J. Arnér, Sel-tag Imaging Project

**Affiliations:** 1 Division of Biochemistry, Department of Medical Biochemistry and Biophysics, Karolinska Institutet, Stockholm, Sweden; 2 Karolinska Experimental Research and Imaging Center, Department of Comparative Medicine, Karolinska University Hospital, Stockholm, Sweden; 3 Department of Clinical Neuroscience, Karolinska Institutet, Stockholm, Sweden; 4 Department of Oncology-Pathology, Cancer Centrum Karolinska, Karolinska Institutet, Stockholm, Sweden; 5 PET Radiochemistry, Neuroradiology Department, Karolinska University Hospital, Stockholm, Sweden; Case Western Reserve University, United States of America

## Abstract

**Background:**

*In vivo* imaging using Annexin A5-based radioligands is a powerful technique for visualizing massive cell death, but has been less successful in monitoring the modest cell death typically seen in solid tumors after chemotherapy. Here we combined dynamic positron emission tomography (PET) imaging using Annexin A5 with a serum-based apoptosis marker, for improved sensitivity and specificity in assessment of chemotherapy-induced cell death in a solid tumor model.

**Methodology/Principal Findings:**

Modest cell death was induced by doxorubicin in a mouse xenograft model with human FaDu head and neck cancer cells. PET imaging was based on ^11^C-labeled Sel-tagged Annexin A5 ([^11^C]-AnxA5-ST) and a size-matched control. 2-deoxy-2-[^18^F]fluoro-D-glucose ([^18^F]-FDG) was utilized as a tracer of tissue metabolism. Serum biomarkers for cell death were ccK18 and K18 (M30 Apoptosense® and M65). Apoptosis in tissue sections was verified *ex vivo* for validation. Both PET imaging using [^11^C]-AnxA5-ST and serum ccK18/K18 levels revealed treatment-induced cell death, with ccK18 displaying the highest detection sensitivity. [^18^F]-FDG uptake was not affected by this treatment in this tumor model. [^11^C]-AnxA5-ST gave robust imaging readouts at one hour and its short half-life made it possible to perform paired scans in the same animal in one imaging session.

**Conclusions/Significance:**

The combined use of dynamic PET with [^11^C]-AnxA5-ST, showing specific increases in tumor binding potential upon therapy, with ccK18/K18 serum measurements, as highly sensitive markers for cell death, enabled effective assessment of modest therapy-induced cell death in this mouse xenograft model of solid human tumors.

## Introduction

Therapy must be individually optimized if outcomes for cancer patients are to be improved [1]. Insufficient induction of cell death in tumors leads to therapeutic failure, while cell death in healthy tissue is the most common dose-limiting side effect. Analyzing the timing, tissue localization and extent of cell death induced by anticancer drugs is therefore necessary for detailed assessments of both therapeutic efficacy and toxic side effects and this could dramatically improve clinical care [2], [3]. Using similar methods for evaluating drug effects in animal models, e.g. mice with xenograft tumors, could reduce the number of animals used to obtain reliable results, improve efficacy read-outs, shorten the time required for translation to the clinic and thereby reduce the costs for drug development. Here we investigated whether positron emission tomography (PET) imaging of cell death in combination with analyses of serum markers could provide a strategy for assessing cell death which is more effective than using either of these techniques alone.

The most widely used cell death imaging methods available to date are based on Annexin A5 (AnxA5), a 36 kDa protein which binds with high affinity to phosphatidylserine (PS) externalized by dying (apoptotic) or dead (late apoptotic or necrotic) cells [2], [3], [4]. ^99m^Tc-HYNIC-AnxA5 is currently in Phase II/III trials [5]. However, AnxA5-based imaging techniques can have difficulty detecting modest responses to therapy such as those often seen clinically in solid tumors. One reason may be that non-specific uptake of protein tracers due to enhanced permeability and retention (EPR), caused by leaky vessels and poor lymphatic drainage in tumors, may mask small variations in specific uptake due to cell death [6], [7]. We have recently developed a new Sel-tag [8], [9], which was used here for ^11^C-labeling of AnxA5. To estimate non-specific tracer uptake due to EPR effects, we also ^11^C-labeled a control ligand, size-matched with AnxA5 but designed to lack probable biological activity in a mammalian host and also shown here not to bind apoptotic cells. We subsequently evaluated the use of these two ligands for PET imaging in a xenograft tumor model, without or with chemotherapy that induces modest cell death. We also compared the results with those of [^18^F]-2-fluoro-2-deoxyglucose ([^18^F]-FDG), a non-metabolized glucose analogue whose uptake is largely proportional to the rate of glucose metabolism [10]. [^18^F]-FDG is currently the PET ligand most widely used in the clinical management of many cancer types, including in the evaluation of therapeutic responses [11].

Monitoring the levels of particular macromolecules released from tumor cells into serum during therapy [12] can, with its ease and low cost, provide an attractive complement to imaging. The cytokeratins, which we monitor here, are useful as circulating biomarkers of epithelial cell death, due to their limited patterns of expression and particularly to the fact that they are not expressed in hematopoietic cells. The release of caspase-cleaved and total keratin 18 (ccK18 and K18, respectively) to serum has been well documented in clinical studies after treatment of breast and prostate cancer patients with cytotoxic drugs [13], [14], [15]. Toxicity to off-target organs, particularly the liver [16], can also give circulating K18 fragments and the extent of these contributions to the serum levels can not be estimated in humans. However, since the antibodies in the M30 Apoptosense and M65 ELISA kits used here are species (human)-specific, serum levels due to effects on human xenografts in rodents will not be affected by cell death in off-target tissues because the murine proteins will not be detected [17].

To be diagnostically useful, methods for assessing cell death must be sufficiently sensitive to detect the levels of responses that occur in clinical situations, i.e. not just the relatively rare instances of complete therapeutic responses with massive apoptosis, which have been more easy to detect by imaging methods [18], [19], but also the partial responses more commonly encountered with solid tumors. Therefore, in order to compare the sensitivities of the PET imaging and serum biomarker methods for assessments of modest tumor cell death and probe their combined usage, we here utilized a squamous carcinoma model that is only moderately sensitive to the drug tested, doxorubicin [20]. We found that the serum biomarker ccK18 proved to be a highly sensitive tool for estimating the extent (but not the localization) of total apoptosis in this model. Dynamic PET imaging for determining the binding potentials of ^11^C-labeled Sel-tagged Annexin A5 ([^11^C]-AnxA5-ST) provided solid information on the cell death in the tumors themselves at discrete time points, particularly when non-specific tumor uptake was estimated using a size-matched control ligand. Therefore we propose that the combined use of serum biomarkers for apoptosis together with dynamic PET imaging using [^11^C]-AnxA5-ST, preferably compared with a size-matched control ligand provides a sensitive technique for detecting, localizing and assessing the extent of cell death induced by chemotherapeutics even in this modestly responsive xenograft model of human solid tumors.

## Materials and Methods

### Materials

If not otherwise specified, all chemicals were obtained from Sigma-Aldrich Chemical Corporation. Specific materials for expression of Sel-tagged proteins were previously described [8], [9], [21]. The cDNA of human Annexin A5 was purchased from OriGene Technologies, Inc. whereas cDNA for GFP was obtained from the pGFPuv plasmid (Clontech).

### Expression and purification of Sel-tagged proteins

A nucleotide sequence encoding a bacterial-type SECIS element and the Sel-tag having the amino acid sequence -Gly-Cys-Sec-Gly was introduced into the Annexin A5 gene by PCR as described [8]. The PCR product was cloned into the *Nde*I-*BamH*I sites of pET-15b (Novagen) and the construct was subsequently transformed into BL21(DE3) *E. coli* strain (Novagen), with the N-terminal His-tag from the vector preserved in the construct for final purification. Protein integrity was also checked by His-tag western blot using SuperSignal West HisProbe Kit (Thermo). The size-matched control protein for assessment of EPR effects was designed to encompass the globular and stable *E. coli* thioredoxin scaffold, made redox inactive by mutating its two active site Cys residues to Ser (mTrx), fused with green fluorescent protein (GFP), which was Sel-tagged (ST) at its C-terminus yielding mTrx-GFP-ST. The resulting DNA sequences of both expression plasmids were verified by DNA sequencing (GATC biotech). The encoded amino acid sequences of the two ligands with their major functional determinants indicated are given in **Material S1**. To increase the yield of Sec insertion into the Sel-tag, the assistant plasmid pSUABC was co-transformed into the expression host bacteria [22] as also previously described in further detail [8]. Upon protein purification based on the N-terminal His-tag of each protein, we included a thorough wash of the nickel column using 50 column volumes of 0.1% triton X-114 before finally eluting the proteins with imidazole, which efficiently removes endotoxins from protein samples [23]. The endotoxin level of different protein samples was subsequently analyzed by the Karolinska Pharmacy and found to be below limits of detection (<2.5 endotoxin units/ml). The purified recombinant proteins were apparently homogenous on Coomassie-stained SDS-PAGE analyses and were obtained at yields of 10–20 mg per liter original bacterial culture, as mixtures of UGA-truncated and Sec-containing full-length proteins, as described [8], with an estimated 20–30% Sec content. They were stored in phosphate-buffered saline at −20°C until use.

### Fluorescence labeling

The AnxA5-ST or mTrx-GFP-ST (5 mg/ml in 100 µL PBS; phosphate buffered saline, 155 mM NaCl, 4 mM phosphate, pH 7.4) were, upon reduction with 2 mM DTT, incubated for 20 min at 20°C with 5 mM 5-iodoacetamidofluorescein (5-IAF, dissolved in DMSO, Invitrogen) for derivatization of the Sec residue in the Sel-tag. The fluorescently labeled proteins, named [AF]-AnxA5-ST and [AF]-mTrx-GFP-ST, were desalted over NAP-5 columns (GE Healthcare) and further purified on an ÄKTAExplorer 10 HPLC system using a Superdex 200 10/300 GL gel filtration column with PBS pH 7.4 as mobile phase. Purity and fluorescence of the proteins were confirmed by SDS-PAGE with UV irradiation and Coomassie staining as described [8], [9], [21].

### Cell culturing

FaDu (human squamous carcinoma) cells (obtained from ATCC, catalogue nr HTB-43) were cultured in Dulbecco's Modified Eagle Medium (D-MEM) with high glucose (Invitrogen) supplemented with 0.1 mM non-essential amino acids, 10% fetal bovine serum (FBS), 1 mM sodium pyruvate, 100 units penicillin/ml, 100 µg streptomycin/ml and 2 mM HEPES at 37°C and 5% CO_2_ in a humidified environment. The integrity of the FaDu cell line was verified with STR profiling cell authentication analysis (LGC Standards).

### Detection of binding to apoptotic cells in culture

FaDu cells were seeded at a density of 100 000 cells/well in 24-well plates 24 h prior to treatment with different concentrations of doxorubicin (0.2 µM, 0.5 µM or 2 µM) for 6, 16, 24 and 48 hours. Upon doxorubicin exposure, cells were washed with PBS and AnxA5 binding buffer (taken from the FITC AnxA5 apoptosis detection kit 556547, BD Pharmingen) before incubation with either 0.5 µg/ml [AF]-AnxA5-ST or [AF]-mTrx-GFP-ST (resuspended in AnxA5 binding buffer) for 20 min at 37°C. Finally the cells were washed with 1× PBS and incubated 5 min with 0.5 µg/ml propidium iodide solution (BD Pharmingen), whereupon pictures were taken using fluorescence microscopy using a Zeiss AxioVert 40CFL microscope.

### Radiolabeling with ^11^C

Radiolabeling of the Sel-tagged proteins was performed in general by the previously published protocol [8]. An aliquot (50 µl) of [^11^C]methyl iodide, prepared according to [24], in DMSO (0.2 ml) was added to the Sel-tagged protein (450 µl, 5 mg/ml) which had been pre-reduced with 2 mM DTT for ≥20 min at room temperature. After reacting for 20 min at 36°C, the product was eluted from a NAP-5 column pre-equilibrated with divalent cation-free PBS. Aliquots of the eluted protein were diluted with saline for immediate injections in the animals. The radiochemical purity was ≥% (Superdex 75 10/300 GL gel filtration column (GE Healthcare) eluted with PBS) and the specific radioactivity 5–10 GBq/µmol protein.

### Ethics statement

This study was performed in strict accordance with the recommendations regarding animal use at Karolinska Institutet and in Sweden. The experiments were approved by the Committee on the Ethics of Animal Experiments of the Northern Stockholm Area (*Stockholms Norra Djurförsöksetiska Nämnd*) and covered in full by the permits with numbers N241/07, N323/09, N85/11, N251/09 and N265/11. Every effort was made to minimize animal suffering and the specific routines of animal handling for the current study is described in the text on methodologies for imaging.

### In vivo PET imaging of xenograft tumors

Animals (SCID mice) were housed under sterile laboratory conditions with food and water ad libitum. FaDu cells (1×10^6^ cells in 0.1 ml PBS) were inoculated subcutaneously into the right flank of each mouse. The mice, bearing tumors of 5 to 9 mm in diameter, were used 1–2 weeks after inoculation. *In vivo* PET studies were performed using a MicroPET Focus 120 (CTI Concorde Microsystems) in general as previously described [25]. The mice were either untreated when scanned or were first injected s.c. with doxorubicin (5 mg/kg) and allowed to recover until the day of the PET imaging. The PET tracer, [^11^C]-AnxA5-ST or [^11^C]-mTrx-GFP-ST (5–9 MBq in 0.2 ml saline), was intravenously injected and PET data were collected for 1 h. At least 30 min after the end of the first scan (allowing decay of low levels of residual ^11^C), either the other ^11^C-labeled protein or [^18^F]-FDG (8–10 MBq, obtained from batches made for clinical PET) was subsequently injected and mice were scanned for an additional 1 h. At the time point for this second injection, the originally injected ^11^C-tracer had decayed to ≈6% of the original injected dose, of which only about 4–6% accumulated per gram of tumor tissue (0.003%). By the last frames of a subsequent [^18^F]-FDG scan, this amount would have decreased through decay to 0.00075% id/g, a level that we concluded, if detectable, was an insignificant contribution to the robust signals from retained [^18^F]-FDG. Immediately after the end of all final scans, the mice were euthanized whereupon tissue and plasma samples were collected for IHC and ccK18 analyses, respectively.

Data were processed using MicroPET Manager and evaluated using the Inveon Resarch Workplace (Siemens Medical Solutions) software. To determine the temporal changes of tracer concentrations, regions of interest (ROIs) were drawn on the images by employing a 75% threshold of the maximum intensity voxels with consistency to post-mortem measurements of the tumor dimensions. Isocontour-delineations employing a standard threshold cut-off are standard techniques for minimizing user bias. Control against the actual size of excised tumor helped ensure that the generated ROIs were representative of the actual tumor volume. Radioactivity concentrations (Bq per milliliter) were calculated automatically by calibrating against a phantom with a known concentration of radioactivity. Assuming a tissue density of 1 g/ml, the radioactivity concentrations were divided by the administered activity to obtain a ROI-derived percent injected dose per gram of tissue (%ID/g). In comparisons between different individuals, normalization to the same body weight was performed.

Data were further analyzed by the graphical method developed by Logan et al. (see [26] and references therein) in combination with the “Simplified Reference Tissue Model” [27]. Here a reference region on, in our case, the quadriceps was also drawn and radioactivity concentrations calculated as above. When fitting a function of the ROI to a function of the reference region, the resulting slope of the curve gives the distribution volume ratio (DVR). By subtracting 1 from the DVR, the binding potential is generated [26], a quantity that is a direct indication of the specific binding sites in the ROI. When analyzed as ratios, compensation against systematic errors is achieved. For visualization purposes, these analyses have been adjusted so they pass through the origin.

### Immunohistochemical analyses

Tumors were resected, fixed in 2% buffered formaldehyde, dehydrated, embedded in paraffin and sectioned. Sections were deparaffinized with xylene, rehydrated, microwaved and then incubated overnight with monoclonal primary antibodies diluted in 1% (wt/vol) bovine serum albumin and visualized using the standard avidin–biotin–peroxidase complex technique (Vector Laboratories, Burlingame, CA, USA). Counterstaining was performed with Mayer's haematoxylin. Antibody against active caspase-3 was from Pharmingen and used at 1∶1000. Scoring of positive cells was performed for three randomly chosen fields of view for each tumor by an independent observer unaware of treatment, who scored the staining as 0–100% positivity. The mean value of this score from the three fields of view was used as the immunohistochemistry score for each tumor.

### Analysis of circulating K18 fragments

Soluble ccK18 and K18 fragments in mouse plasma were determined by the M30 Apoptosense® and M65® ELISA assays [13], [17] (Peviva AB, Bromma, Sweden) (note: even though plasma was used in the present study the analyte is generally referred to as a “serum biomarker” in the literature and we have chosen to do so here). M30 Apoptosense® is a sandwich immunoassay based on two monoclonal antibodies: M30 and M5. M30 recognizes a K18 neo-epitope generated by caspase cleavage during apoptosis, but does not recognize uncleaved K18. M5 recognizes an epitope in the 284–396 region of K18. The M65 ELISA® measures total soluble K18 fragments using the M5 and M6 monoclonal antibodies and measures total epithelial cell death [17]. Neither M30 nor M5 recognize mouse K18; the M30 Apoptosense® and M65® ELISA assays are therefore specific for tumor apoptosis/cell death when plasma is analyzed from mice carrying human tumor xenografts [17]. The ELISA's were used according to the instructions of the manufacturer (except that 12.5 µl plasma sample was used). HBR-Plus blocking reagent was used to reduce background observed in mouse plasma (lot#5130, conc. 10.6 mg/ml; 3KC579; Scantibodies laboratory Inc, Santee CA, USA), 0.4 µl of per sample

### Statistical methods

Data from biomarker analyses (IHC scores, M30 Apoptosense and binding potentials in PET imaging) were presented as median values. Analysis of correlation was performed using the Spearman nonparametric test (Prism software). To test for statistically significant differences between any two groups, the nonparametric Mann-Whitney U method not assuming Gaussian distribution was used (Prism software). All tests were two-tailed.

## Results

### Induction of cell death by doxorubicin in FaDu tumor xenograft tissue

Human FaDu head-neck carcinoma shows a modest response to DNA damaging agents, resulting in tumor growth retardation but not in eradication [20]. Thus these cells were used here in mouse xenografts to model clinical reality and to challenge the levels of sensitivity for the cell death detection methods.

**Figure 1:**
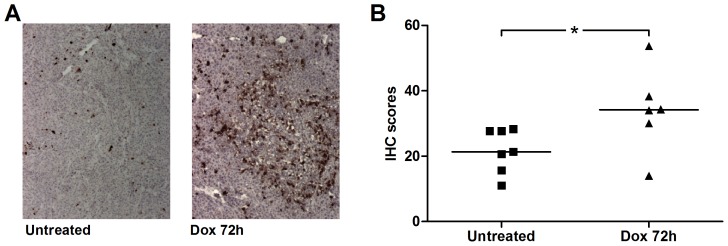
Assessment of tumor cell death using immunohistochemistry. ***A*** *)* Illustrative immunohistochemical analyses of active caspase-3 (staining dark brown) in tumor tissue before (left) or 72 h after treatment with a single dose doxorubicin (right), showing increased tumor apoptosis *in situ*. ***B*** *)* Summary of immunohistochemistry (IHC) scores in FaDu xenografts analyzed postmortem with (triangles, *n* = 6) or without (squares, *n* = 7) prior doxorubicin treatment for 72 h. Vertical line shows median and the single asterisk indicates the statistical significance between the two groups (*p = 0.0381*). Each tumor score represents the mean score from analyses of three separate fields of view per tumor evaluated by a person unaware of treatment and setting an arbitrary score between 0 (no apparent staining) to 100 (all cells staining positive).

First the extent of cell death provoked by a single dose of doxorubicin in the FaDu xenograft tumors was determined by staining sections of excised tumors from non-treated and treated animals with an antibody recognizing active caspase-3. Basal levels of apoptosis were observed in tumors from non-treated mice and these levels were increased in tumors from animals excised 72 hours after doxorubicin treatment as evaluated by this immunohistochemical approach (**Fig. 1A**). The patterns of apoptosis were, however, heterogeneous in the tissue sections and were in some sections similar to that in the untreated control. The statistical analyses of the immunohistochemictry scoring results confirmed the induction of a modest increase in apoptosis using this treatment protocol (**Fig. 1B**).

### PET Imaging using [^11^C]-AnxA5-ST and a size-matched control ligand

Evaluation of therapeutic responses by imaging has the advantages that it is non-invasive, provides positional information and samples over the entire tumor (as opposed to sectioning for immunohistochemistry). For imaging tumor cell death we here used [^11^C]-AnxA5-ST. To assess passive tissue uptake of protein tracer into tumor tissue due to EPR effects [6], [7], we used the control ligand [^11^C]-mTrx-GFP-ST that is presumed to lack biological effects in a mammalian host (for details see *Materials and Methods* and **Material S1**). First we verified that fluorescently labeled AnxA5-ST, but not mTrx-GFP-ST, bound to doxorubicin-treated FaDu cells in culture (**Fig. S1**). Thereafter the two ligands were site-specifically ^11^C-labeled at the Sec residue in their Sel-tag motifs and used for dynamic PET imaging with the FaDu cell xenograft-bearing mice.

**Figure 2:**
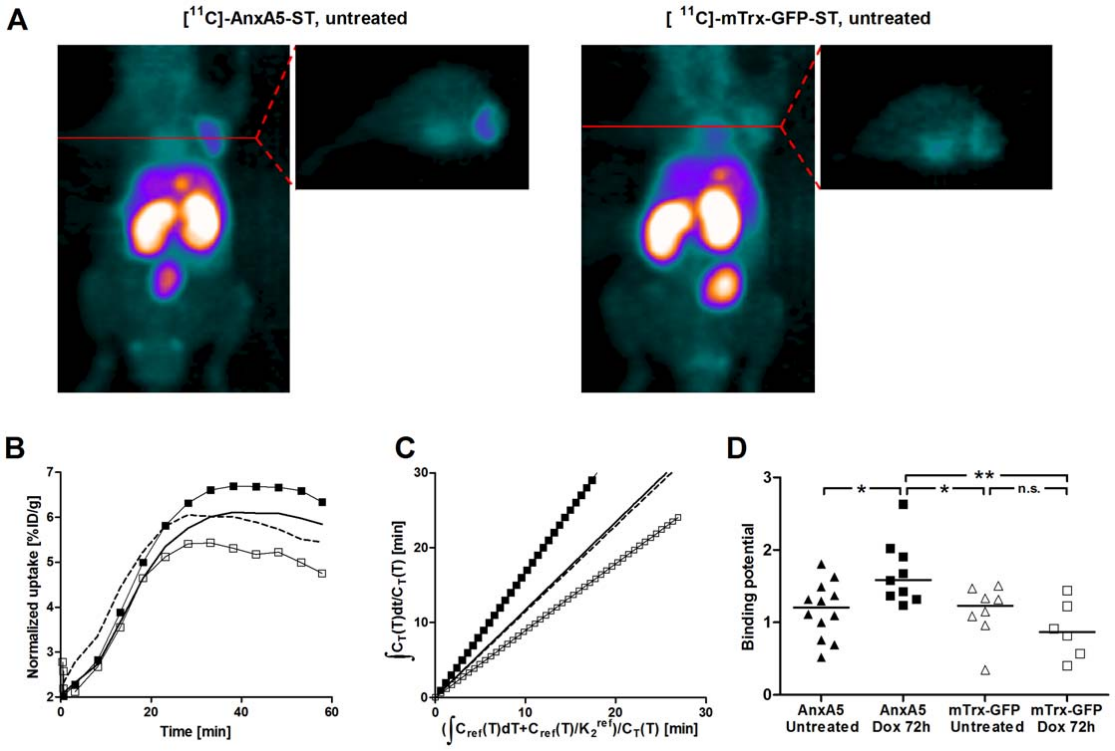
Increased tumor uptake upon doxorubicin treatment using [^11^C]-AnxA5-ST but not [^11^C]-mTrx-GFP-ST. ***A*** *)* MIP and transaxial images of radioactivity (summed over 10–55 min) after tail vein injection of [^11^C]-AnxA5-ST (left) or [^11^C]-mTrx-GFP-ST (right) in a mouse bearing a FaDu xenograft without doxorubicin treatment. ***B*** *)* Time activity curves for tumor uptake of [^11^C]-AnxA5-ST (solid squares or solid line) and [^11^C]-mTrx-GFP (empty squares or dashed line) in untreated mice (lines) or 72 h after a single-dose treatment with doxorubicin (squares). Note the similar uptake of [^11^C]-AnxA5-ST and [^11^C]-mTrx-GFP-ST in untreated mice, due to the non-specific EPR effect, while upon doxorubicin treatment an increase in [^11^C]-AnxA5-ST uptake was seen in contrast to a non-statistically significant tendency to decrease of [^11^C]-mTrx-GFP-ST. ***C*** *)* Logan plot analyses of distribution volumes of [^11^C]-AnxA5-ST or [^11^C]-mTrx-GFP-ST in FaDu xenograft tumors in untreated and doxorubicin treated mice (symbols as in panel *B*). ***D*** *)* Diagram summarizing tumor binding potential in PET imaging using either [^11^C]-AnxA5-ST (solid symbols) or the size-matched control protein [^11^C]-mTrx-GFP-ST (open symbols) with the mice carrying FaDu xenograft tumors, either with (squares, *n* = 9 for [^11^C]-AnxA5-ST and *n* = 6 for [^11^C]-mTrx-GFP-ST) or without (triangles, *n* = 12 for [^11^C]-AnxA5-ST and *n* = 8 for [^11^C]-mTrx-GFP-ST), with asterisks indicating statistical significance between pair of groups. The *p* values were as follows: [^11^C]-AnxA5-ST in untreated tumors *vs.* the same ligand in doxorubicin treated: *p = 0.0173*; [^11^C]-AnxA5-ST in doxorubicin treated tumors *vs.* [^11^C]-mTrx-GFP-ST in untreated tumors: *p = 0.0152*; [^11^C]-AnxA5-ST in doxorubicin treated tumors *vs.* [^11^C]-mTrx-GFP-ST in doxorubicin treated tumors: *p = 0.0048*; remaining pair-wise comparisons showed a lack of statistically significant differences, as indicated for [^11^C]-mTrx-GFP-ST with or without treatment (n.s. = no significance, *p>0.1*).

Graphical analyses for the quantification of uptake of radioactivity in tissues performed as described [26], [27], [28] revealed that the uptake from [^11^C]-AnxA5-ST and [^11^C]-mTrx-GFP-ST were similar in untreated human FaDu cell xenograft tumors (**Figs 2A, 2B**), suggesting that these basal levels were largely due to passive uptake. At 72 h after a single dose of doxorubicin (5 mg/kg s.c.), [^11^C]-AnxA5-ST uptake in tumors was elevated compared to the untreated tumors. The treatment-induced increases were of a magnitude consistent with the levels of increased caspase-positivity in tissue sections (**Fig. 1**). Changes in the distribution volume ratio, as illustrated in Logan plots [28], provided robust read-outs for the increases of [^11^C]-AnxA5-ST uptake (**Fig. 2C**). In contrast, doxorubicin treatment did not increase the distribution volume ratio for the control ligand [^11^C]-mTrx-GFP-ST (**Fig. 2C**). Imaging showed a statistically significant increase in the tumor binding potential of [^11^C]-AnxA5-ST after doxorubicin, which was even larger if compared to [^11^C]-mTrx-GFP-ST (**Fig. 2D**). Distribution volume ratios assessed at 48 h after a lower dose of doxorubicin (2.5 mg/kg s.c.) were in between the baseline levels and those after the higher dose at 72 h (**Fig. S2**).

### PET imaging with either [^11^C]-AnxA5-ST or [^11^C]-mTrx-GFP-ST compared to [^18^F]-FDG

**Figure 3:**
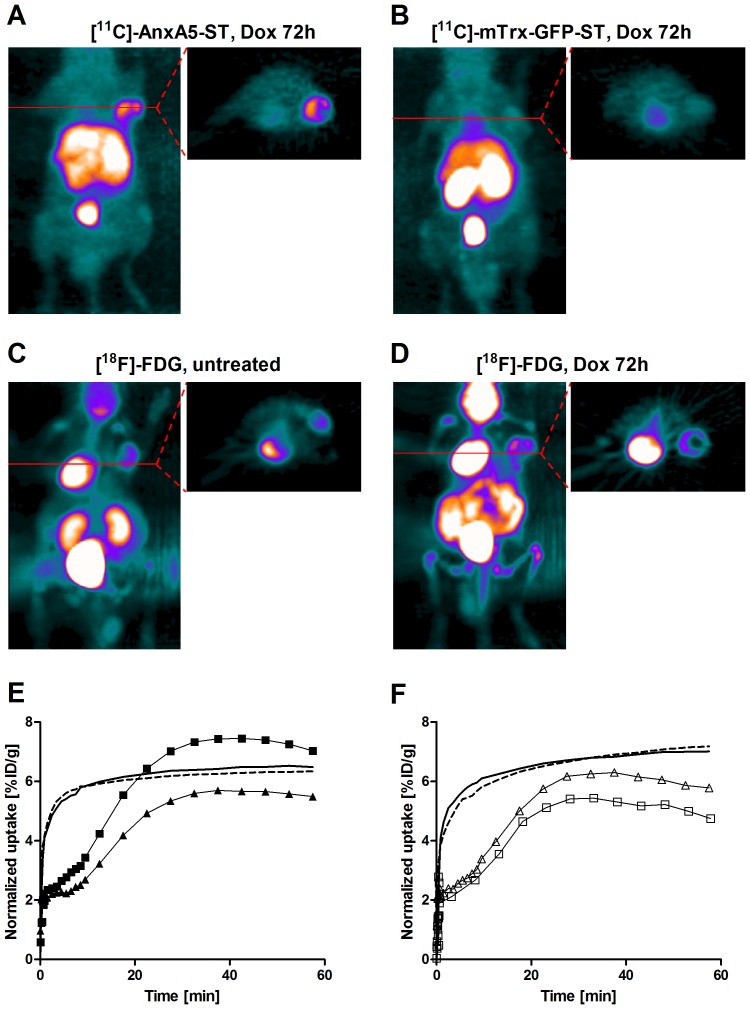
Response to doxorubicin at 72 h is revealed with [^11^C]-AnxA5-ST but not [^18^F]-FDG or [^11^C]-mTrx-GFP-ST. Representative MIP and transaxial images of radioactivity (summed over 35–55 min) after tail vein injection in mice with FaDu xenografts of ***A*** *)* [^11^C]-AnxA5-ST or ***B*** *)* [^11^C]-mTrx-GFP-ST, 72 h after treatment with a single dose of doxorubicin (5 mg/kg). Also shown are representative images of [^18^F]-FDG, at ***C*** *)* baseline or, alternatively, ***D*** *)* after treatment with doxorubicin and 90 min after the image with [^11^C]-AnxA5-ST in ***A*** *)*. ***E*** *)* Time activity curves of single tumors showing uptake of [^11^C]-AnxA5-ST (solid triangles and squares) or [^18^F]-FDG (lines) before (solid triangle and dashed line) or after doxorubicin treatment (solid squares and solid line). ***F*** *)* Corresponding experiments showing a comparison between of [^11^C]-mTrx-GFP-ST (empty triangles and squares) and [^18^F]-FDG (lines) tumor uptake in the same mouse before (empty triangles and dashed line) or after doxorubicin treatment (empty squares and solid line).

The short half-life of ^11^C allowed scans with [^18^F]-FDG (**Fig. 3C, 3D**) to be performed within a few hours after imaging with either [^11^C]-AnxA5-ST or [^11^C]-mTrx-GFP-ST. [^18^F]-FDG was more rapidly taken up in the tumor in baseline and treated animals compared to the protein ligands (cf. **Fig. 2A, 2B** **and** **Fig. 3A, 3B, 3C, 3D, 3F**), consistent with a more rapid distribution of small water-soluble carbohydrates from blood into tissues. However, in contrast to the drug-induced increases in [^11^C]-AnxA5-ST uptake, the tumor uptake of [^18^F]-FDG was unchanged at 72 hrs after the doxorubicin treatment, as compared to untreated mice (**Fig. 3E**) or compared to [^11^C]-mTrx-GFP-ST (Fig. **3F**).

### Increased levels of circulating cell death biomarkers

**Figure 4:**
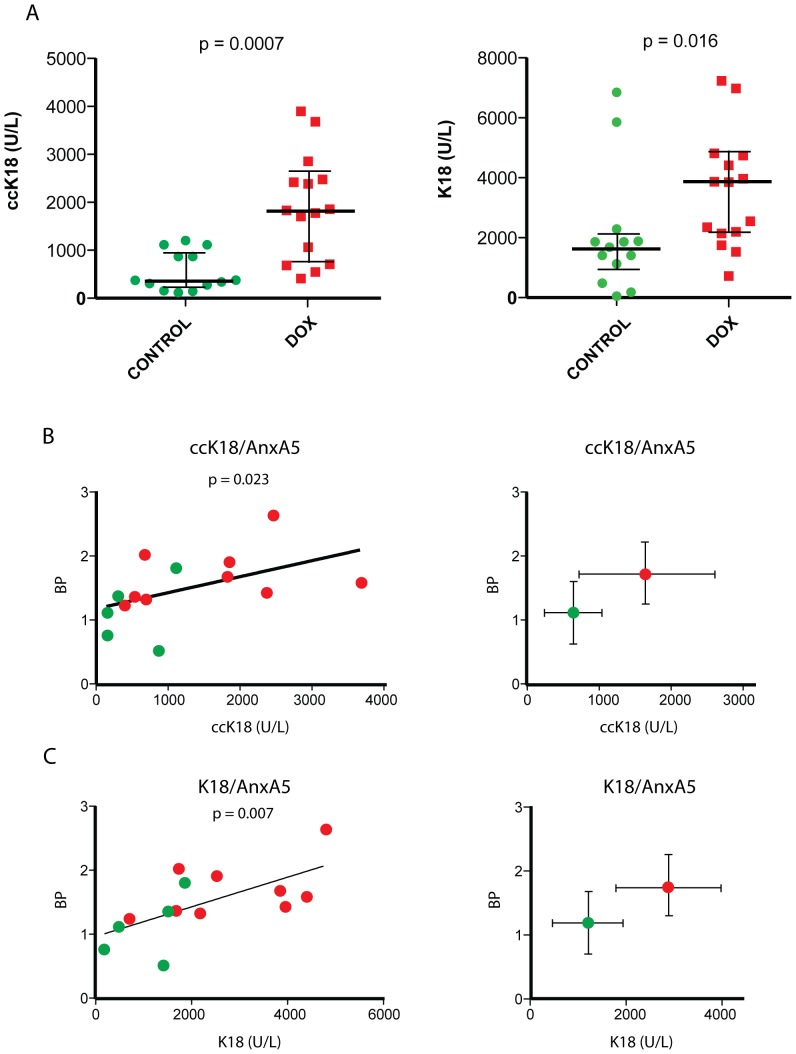
Correlations between increases in serum marker levels and tumor [^11^C]-AnxA5-ST binding potentials upon doxorubicin treatment. ***A*** *)* Levels of ccK18 (M30 Apoptosense®) and K18 (M65®) determined in plasma of mice carrying FaDu xenograft tumors, either with (red symbols) or without (green symbols) prior doxorubicin treatment for 72 h. Statistical significance as given in the panels was determined by the Mann-Whitney *U* test. ***B,C*** *)* Correlations between tumor binding potential in PET imaging using the [^11^C]-AnxA5-ST tracer and ***B*** *)* ccK18 and ***C*** *)* K18 plasma levels for each mouse. Shown to the left in each panel are values of individual mice with the p-values of statistical analysis of correlation as performed using the Spearman rank test given in the graphs. The plots to the right show the aggregated means ± S.D. Red symbols: doxorubicin-treated mice; green symbols: untreated mice.

We found that the plasma levels of caspase-cleaved K18 (ccK18) were significantly increased when sampled 72 hours after doxorubicin treatment as compared to untreated tumor-bearing mice (*p = 0.0007*, **Fig. 4A**). The increases were larger than expected from *in situ* detection of apoptosis by staining for active caspase-3 and larger than those observed during PET imaging with [^11^C]-AnxA5-ST. We furthermore determined the levels of human circulating total K18 using the M65 ELISA, a biomarker for total cell death [13], which also showed a pronounced increase in levels comparing treated animals with controls (*p = 0.016*, **Fig. 4A**). Assessment of active caspase-3 by immunohistochemistry showed considerable variability between individual sections from the same tumor (**Fig. S3**). This is in contrast to imaging and serum biomarker analyses, which will reflect apoptosis/cell death of the entire tumor mass. Indeed, when examining correlations between the tumor binding potentials of the [^11^C]-AnxA5-ST tracer and levels of circulating ccK18 fragments in tumor-bearing animals, these were found to be significant (*p = 0.023* by Spearman rank correlation, **Fig. 4B**), as they also were between [^11^C]-AnxA5-ST binding potentials and plasma K18 (p = 0.007, **Fig. 4C**). This suggested that both the [^11^C]-AnxA5-ST tumor binding potentials and ccK18 levels in plasma could be used as reliable biomarkers for therapy-induced cell death. In contrast, there was no correlation between [^11^C]-mTrx-GFP-ST binding potentials and either ccK18 or K18 (*p = 0.50* and *p = 0.87*, respectively). Although both the serum markers and PET imaging of tumor uptake with [^11^C]-AnxA5-ST displayed statistically significant increases with treatment, the serum markers gave higher magnitude in their increases and would thereby likely show better sensitivity in cell death detection (cf. **Fig. 2C** and **Fig. 4A**). The fold changes in median values after doxorubicin treatment were about 1.5-fold higher for the binding potentials of [^11^C]-AnxA5-ST whereas they were about 5-fold higher for the ccK18 marker (see **Fig. S4** for a summary of the fold changes). However, in spite of its higher sensitivity the ccK18 marker can, naturally, not localize the site of its generation. We therefore conclude that the combination of PET imaging with [^11^C]-AnxA5-ST, to reveal the localization of cell death, with determination of the serum ccK18 (M30) biomarker, as a measure of accumulated cell death, should be a promising method for assessment of therapeutic efficacy.

## Discussion

Assessment of whether drug treatment induces cell death in solid tumors when using biopsy material is complicated by the fact that the levels observed in tissue sections are variable, both at base-line and following therapy. This variability is likely to reflect tumor heterogeneity and the presence of different clones in the tumor, showing different rates of spontaneous cell death and different sensitivities to the drug used. Assessment of treatment responses therefore requires both paired tumor samples (before and after therapy) and several biopsy cores. Pharmacokinetics of a chemotherapeutic drug as well as the inherent kinetics of cell death processes clearly also affect the results at any given time point. Imaging techniques are hence powerful alternatives as they may integrate the overall response of a tumor and can therefore be less affected by tumor heterogeneity at a microscopic level, while still providing positional information. Imaging methods also have the capacity to detect off-target toxicity to non-tumor tissue. Other factors, however, complicate tumor cell death imaging, such as the phenomenon of non-specific uptake of tracer in tumor tissue due to EPR effects [7] and possible effects of anti-cancer drugs on the vasculature thereby affecting tracer deposition. Using [^11^C]-mTrx-GFP-ST to examine EPR effects we found a tendency for decrease in uptake upon doxorubicin treatment at 72 hours, even though it was not statistically significant. Doxorubicin has indeed been reported to affect endothelial cell function [29] and the actual imaging read-out for assessment of the AnxA5-specific uptake should therefore be the difference between [^11^C]-AnxA5-ST and [^11^C]-mTrx-GFP-ST uptakes after treatment, rather than [^11^C]-AnxA5-ST alone compared to baseline. Whether changes in passive tracer uptake have influenced abilities of other studies with AnxA5 ligands to reveal therapy-induced cell death is unclear, but can not be excluded. To our knowledge, this is the first PET study comparing a size-matched ligand with AnxA5 to discriminate different chemotherapy effects in a tumor xenograft model using PET imaging.

In the FaDu tumor model used here, the amplitude of increased uptake of [^11^C]-AnxA5-ST following doxorubicin treatment was similar to that which could be expected from the extent of increase in the number of apoptotic cells in tissue at that time point. Interestingly, the increases in the levels of the serum K18 biomarkers – and in particular ccK18 (the M30 ELISA analyte) - were clearly larger than the increases in tumor binding potential for [^11^C]-AnxA5-ST. A possible explanation for the strong increases in circulating K18 molecules should be that they are accumulated in the blood after instigation of apoptosis [12]. The higher signals increase sensitivity in detection for the entire time interval since start of treatment, but serum biomarkers have the disadvantage of not providing regional information about the source of the cell death signal. We therefore believe that the combination of sensitive serum cell death biomarkers with *in vivo* tissue cell death imaging using PET should be attractive for accurately determining treatment responses as well as toxic side effects to off-target tissues.

In conclusion, our results illustrate the feasibility of studying cell death through multimodality and multi-tracer imaging strategies utilizing ^11^C-labeled Sel-tagged proteins. Comparing the uptake of AnxA5 with a size-matched control and using dynamic imaging protocols were fundamentally important for revealing the therapy-induced responses. We propose that these imaging biomarkers can provide information about therapeutic responses, as here to doxorubicin, that is discrete from the metabolic read-outs obtained with the more widely used imaging biomarker [^18^F]-FDG. Furthermore, complementing the imaging with measurements of the circulating serum biomarkers of cell death, ccK18/K18, increases the total sensitivity of the detection. Since both methods are comparatively rapid and used with live mice, this approach provides a promising platform for determinations of both kinetics and tissue specificities in cell death detection as well as potential multiple effects of the therapeutics during drug development using xenograft tumor models in mice.

## Supporting Information

Material S1 Detailed description of the new AnxA5-ST and mTrx-GFP-ST PET ligands, including amino acid sequences and description of functional domains.
(DOCX)

Figure S1 **AnxA5-ST maintains specific binding to apoptotic cells when labeled with fluorescence at its Sec residue whereas mTrx-GFP-ST does not bind apoptotic cells.** Fluorescence microscopy (top) or phase contrast (bottom) pictures of FaDu cells exposed to 0.2 µM doxorubicin for 48 hours and incubated with 0.5 µg/ml of either [AF]-AnxA5-ST (left) or [AF]-mTrx-GFP (right) for 20 min at 37°C. Fluorescence labeling at the Sel-tag was performed as described in the *Materials and Methods* section.
(JPEG)

Figure S2 **Uptake of [^11^C]-AnxA5-ST in tumors with two separate doxorubicin treatment regimes.** Logan plots are shown for [^11^C]-AnxA5-ST tumor uptake in untreated controls or after 48-hour doxorubicin treatment (2.5 mg/kg) or 72-hour treatment (5 mg/kg), compared to [^11^C]-mTrx-GFP-ST in either untreated tumors or after 72-hour doxorubicin treatment (5 mg/kg), as indicated. The binding potential of [^11^C]-AnxA5-ST was, as expected, intermediate using the intermediate dose. This dose was not analyzed using [^11^C]-mTrx-GFP-ST. For further analyses see Supplementary Fig. S3 and the main text of the study.
(PDF)

Figure S3 **Illustration of variability between tumors and tumor sections receiving the same treatment and comparisons between [^11^C]-AnxA5-ST imaging, active caspase-3 staining and M30/M65 scores for the same tumors.** Two illustrative examples are shown each for tumors of untreated mice (top), 48-hour doxorubicin treatment (2.5 mg/kg) or 72-hour treatment (5 mg/kg), with PET image of the tumor using [^11^C]-AnxA5-ST shown to the left, three sections of each tumor to the right (with IHC scores) and a summary of the data including M30/M65 scores to the left. See text for further details.
(JPEG)

Figure S4 **Fold changes upon doxorubicin treatment in the signal of biomarkers studied herein.** This bar graph illustrates the fold changes in biomarker signals detected upon 72-hour after a single-dose doxorubicin treatment (5 mg/kg). The bars illustrate the ratio of median values for treated animals over controls, with binding potentials determined through Logan plots for [^11^C]-AnxA5-ST or [^11^C]-mTrx-GFP-ST as well as the M30 or M65 serum markers. For further details please see the main text of the study.
(JPEG)
