# Supplementary material for: Combining [11C]-AnxA5 PET Imaging with Serum Biomarkers for Improved Detection in Live Mice of Modest Cell Death in Human Solid Tumor Xenografts
Source: PLoS One. 2012 Aug 1;7(8):e42151. doi: 10.1371/journal.pone.0042151 (PMC3411630; doi:10.1371/journal.pone.0042151)
Supplement: Material S1 — Detailed description of the new AnxA5-ST and mTrx-GFP-ST PET ligands, including amino acid sequences and description of functional domains. (DOCX) [file pone.0042151.s001.docx]

Combining [^11^C]-AnxA5 PET imaging with Serum Biomarkers for Improved Detection in Live Mice of Modest Cell Death in Human Solid Tumor Xenografts

Qing Cheng^1^, Li Lu^2,3^, Jonas Grafström^3^, Maria Hägg Olofsson^4^, Jan-Olov Thorell^3,5^, Erik Samén^3,5^, Katarina Johansson^1^, Hanna-Stina Ahlzén^1^, Sharon Stone-Elander^1,3,5^, Stig Linder^4^ and Elias S J Arnér^1,§^ for the Sel-tag imaging project^¶^

**Supplementary material S1:** Detailed description of the new AnxA5-ST and mTrx-GFP-ST PET ligands, including amino acid sequences and description of functional domains.

The Sel-tagged AnxA5 (AnxA5-ST) and a size-matched control protein designed to lack presumed biological activity (mTrx-GFP-ST) as ligands for PET imaging based upon 11C-labeling at the Sec residue, or which could alternatively also be labeled fluorescently with 5-IAF, were produced and purified as described in *Materials and Methods* of the main text. Below is the amino acid sequences given of the two ligands as designed and verified by DNA sequence determination of the corresponding plasmids, with the key features of each protein indicated.

**AnxA5-ST**

MGSSHHHHHHSSGLVPRGSHMAQVLRGTVTDFPGFDERADAETLRKAMKGLGTDEESILTLLTSRSNAQRQEISAAFKTLFGRDLLDDLKSELTGKFEKLIVALMKPSRLYDAYELKHALKGAGTNEKVLTEIIASRTPEELRAIKQVYEEEYGSSLEDDVVGDTSGYYQRMLVVLLQANRDPDAGIDEAQVEQDAQALFQAGELKWGTDEEKFITIFGTRSVSHLRKVFDKYMTISGFQIEETIDRETSGNLEQLLLAVVKSIRSIPAYLAETLYYAMKGAGTDDHTLIRVMVSRSEIDLFNIRKEFRKNFATSLYSMIKGDTSGDYKKALLLLCGEDDGC**U**G

Molecular Weight = 38467.262

Extinction Coefficient = 21050 M^-1^cm^-1^

N-terminal His-tag: Shadowed

AnxA5: Yellow

Sel-tag (ST): Red

Selenocysteine residue (Sec/U): Bold

**mTrx-GFP-ST**

MGHHHHHHLSDKIIHLTDDSFDTDVLKADGAILVDFWAEWSGPSKMIAPILDEIADEYQGKLTVAKLNIDQNPGTAPKYGIRGIPTLLLFKNGEVAATKVGALSKGQLKEFLDANLAGSGSGHMSKGEELFTGVVPILVELDGDVNGHKFSVSGEGEGDATYGKLTLKFICTTGKLPVPWPTLVTTFSYGVQCFSRYPDHMKRHDFFKSAMPEGYVQERTISFKDDGNYKTRAEVKFEGDTLVNRIELKGIDFKEDGNILGHKLEYNYNSHNVYITADKQKNGIKANFKIRHNIEDGSVQLADHYQQNTPIGDGPVLLPDNHYLSTQSALSKDPNEKRDHMVLLEFVTAAGITHGMDELYKGC**U**G

Molecular Weight = 40423.609

Extinction Coefficient = 33710 M^-1^cm^-1^

N-terminal His-tag: Shadowed

mTrx: Blue, with its two active site Cys residues mutated to Ser (underlined)

GFP (GFPuv): Green

Sel-tag (ST): Red

Selenocysteine residue (Sec/U): Bold
