# Supplementary figures and images for: Combining [11C]-AnxA5 PET Imaging with Serum Biomarkers for Improved Detection in Live Mice of Modest Cell Death in Human Solid Tumor Xenografts
Source: PLoS One. 2012 Aug 1;7(8):e42151. doi: 10.1371/journal.pone.0042151 (PMC3411630; doi:10.1371/journal.pone.0042151)

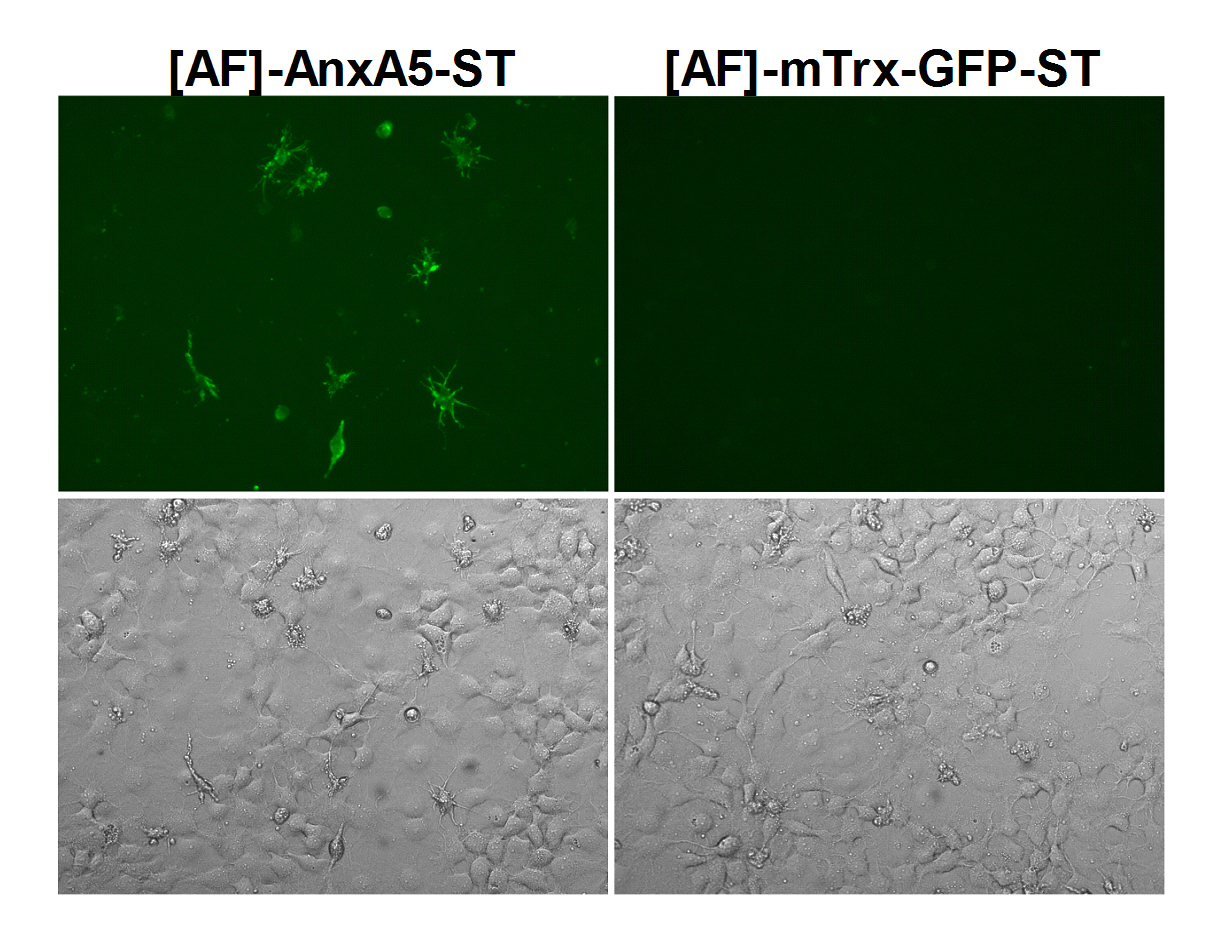

Supplement: Figure S1 — AnxA5-ST maintains specific binding to apoptotic cells when labeled with fluorescence at its Sec residue whereas mTrx-GFP-ST does not bind apoptotic cells. Fluorescence microscopy (top) or phase contrast (bottom) pictures of FaDu cells exposed to 0.2 µM doxorubicin for 48 hours and incubated with 0.5 µg/ml of either [AF]-AnxA5-ST (left) or [AF]-mTrx-GFP (right) for 20 min at 37°C. Fluorescence labeling at the Sel-tag was performed as described in the Materials and Methods section. (JPEG) [file pone.0042151.s002.jpeg]

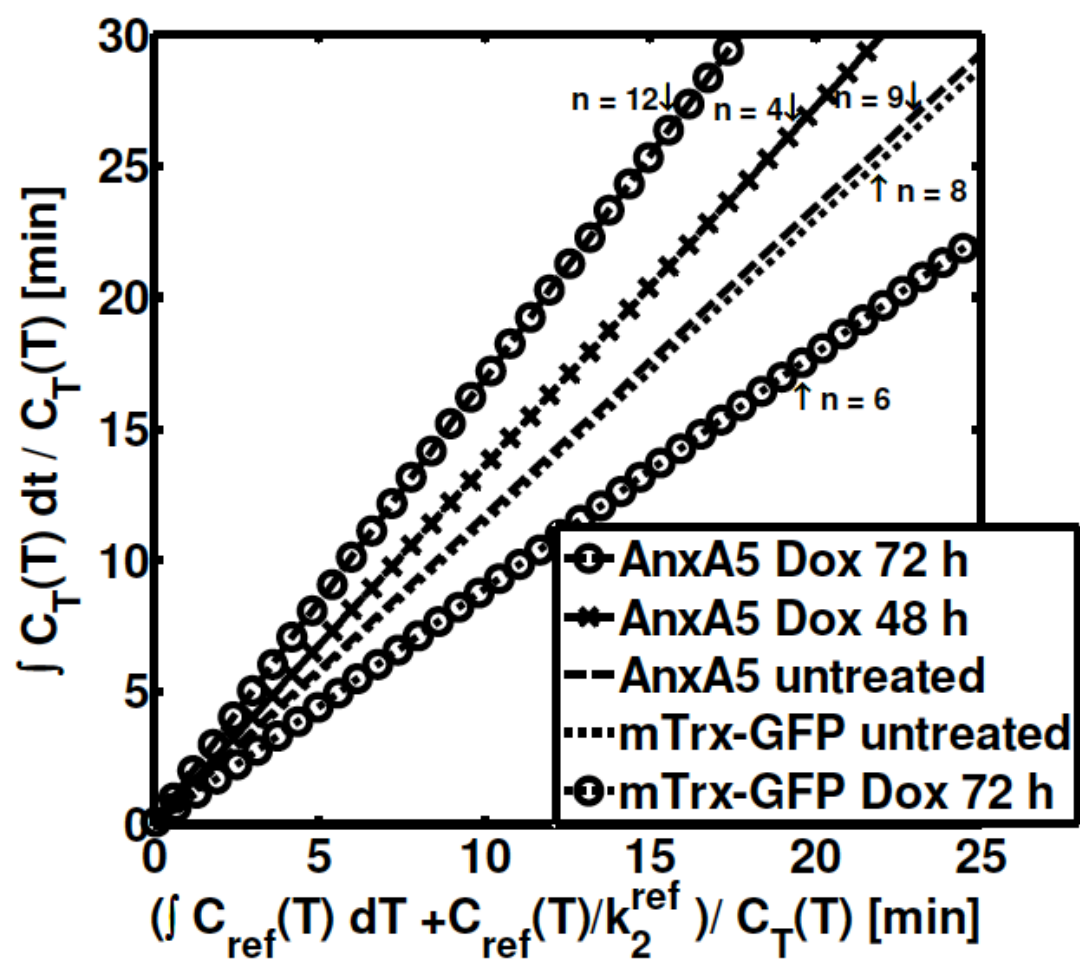

Supplement: Figure S2 — Uptake of [11C]-AnxA5-ST in tumors with two separate doxorubicin treatment regimes. Logan plots are shown for [11C]-AnxA5-ST tumor uptake in untreated controls or after 48-hour doxorubicin treatment (2.5 mg/kg) or 72-hour treatment (5 mg/kg), compared to [11C]-mTrx-GFP-ST in either untreated tumors or after 72-hour doxorubicin treatment (5 mg/kg), as indicated. The binding potential of [11C]-AnxA5-ST was, as expected, intermediate using the intermediate dose. This dose was not analyzed using [11C]-mTrx-GFP-ST. For further analyses see Supplementary Fig. S3 and the main text of the study. (PDF) [file pone.0042151.s003.pdf]

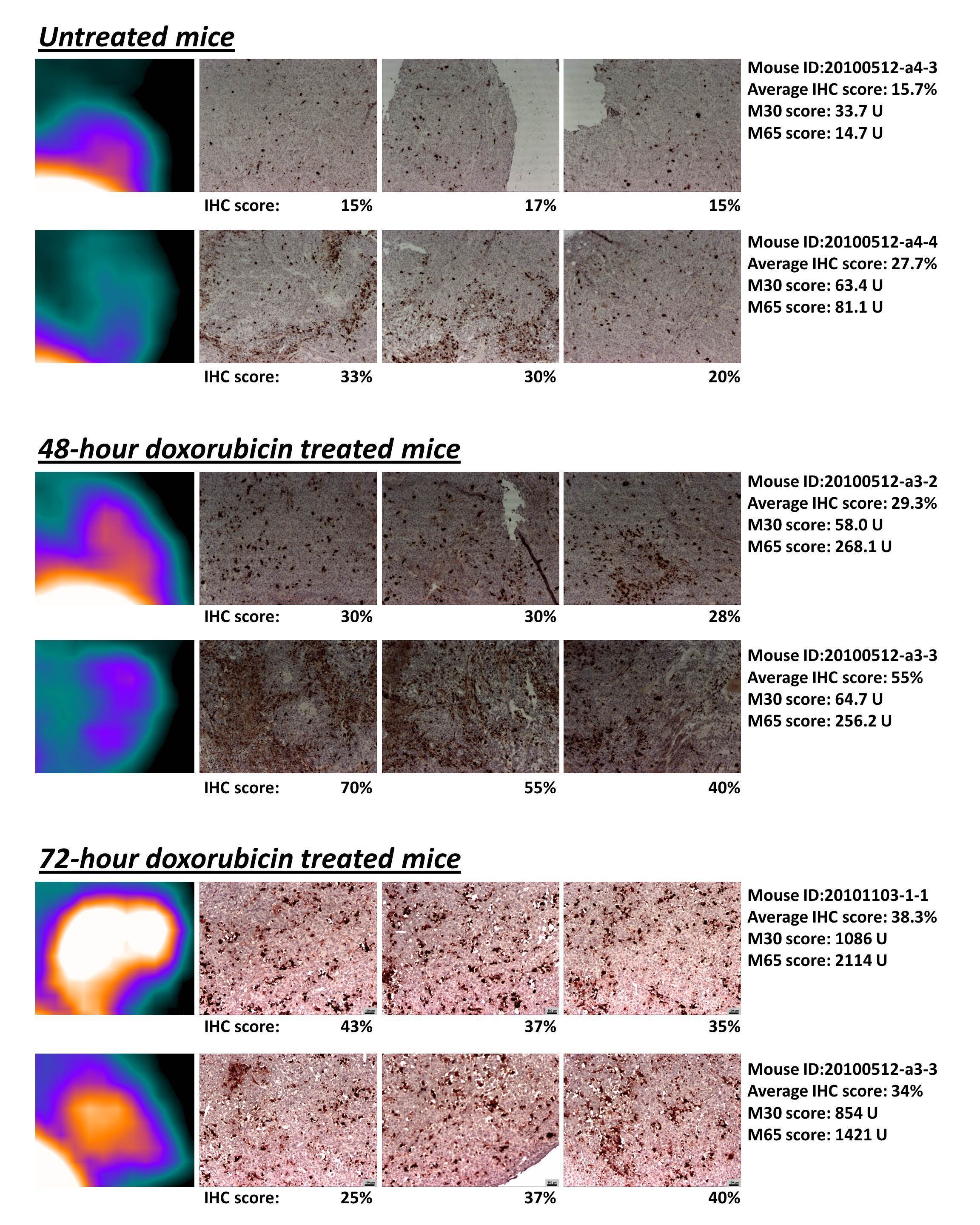

Supplement: Figure S3 — Illustration of variability between tumors and tumor sections receiving the same treatment and comparisons between [11C]-AnxA5-ST imaging, active caspase-3 staining and M30/M65 scores for the same tumors. Two illustrative examples are shown each for tumors of untreated mice (top), 48-hour doxorubicin treatment (2.5 mg/kg) or 72-hour treatment (5 mg/kg), with PET image of the tumor using [11C]-AnxA5-ST shown to the left, three sections of each tumor to the right (with IHC scores) and a summary of the data including M30/M65 scores to the left. See text for further details. (JPEG) [file pone.0042151.s004.jpeg]

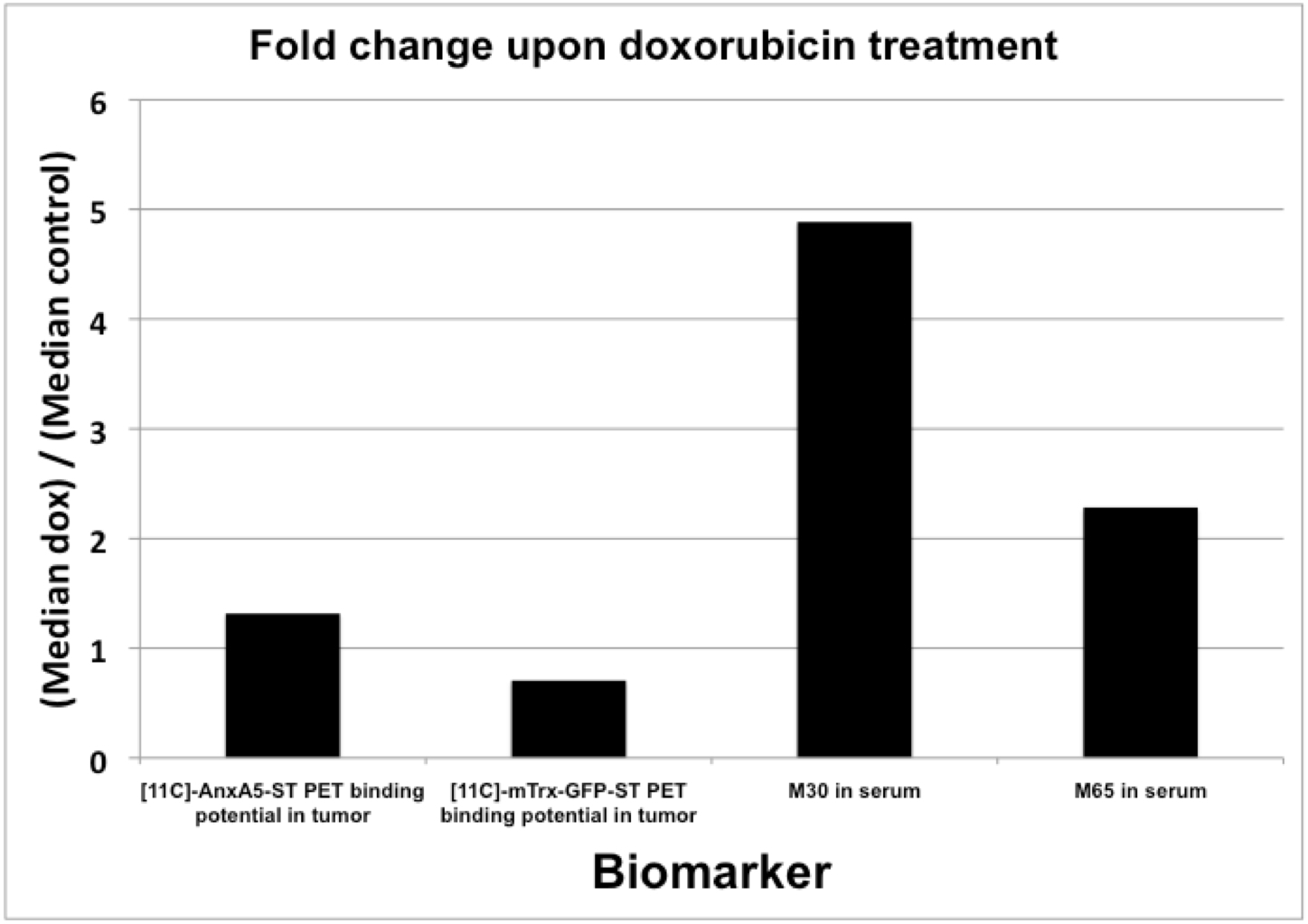

Supplement: Figure S4 — Fold changes upon doxorubicin treatment in the signal of biomarkers studied herein. This bar graph illustrates the fold changes in biomarker signals detected upon 72-hour after a single-dose doxorubicin treatment (5 mg/kg). The bars illustrate the ratio of median values for treated animals over controls, with binding potentials determined through Logan plots for [11C]-AnxA5-ST or [11C]-mTrx-GFP-ST as well as the M30 or M65 serum markers. For further details please see the main text of the study. (JPEG) [file pone.0042151.s005.jpeg]
